# Role of HIV Infection Duration and CD4 Cell Level at Initiation of Combination Anti-Retroviral Therapy on Risk of Failure

**DOI:** 10.1371/journal.pone.0075608

**Published:** 2013-09-24

**Authors:** Sara Lodi, Andrew Phillips, Sarah Fidler, David Hawkins, Richard Gilson, Ken McLean, Martin Fisher, Frank Post, Anne M. Johnson, Louise Walker-Nthenda, David Dunn, Kholoud Porter, on behalf of the UK Register of HIV

**Affiliations:** 1 Medical Research Council, University College London, London, United Kingdom; 2 Instituto de Salud Carlos III, Madrid, Spain; 3 University College London, London, United Kingdom; 4 Imperial College NHS Trust, London, United Kingdom; 5 Chelsea and Westminster Hospital, London, United Kingdom; 6 Mortimer Market Centre and University College London Hospitals, London, United Kingdom; 7 Charing Cross Hospital, London, United Kingdom; 8 Brighton and Sussex University Hospital National Health Service Trust, Brighton, United Kingdom; 9 King’s College, London, United Kingdom; Institute of Infectious Diseases and Molecular Medicine, South Africa

## Abstract

**Background:**

The development of HIV drug resistance and subsequent virological failure are often cited as potential disadvantages of early cART initiation. However, their long-term probability is not known, and neither is the role of duration of infection at the time of initiation.

**Methods:**

Patients enrolled in the UK Register of HIV seroconverters were followed-up from cART initiation to last HIV-RNA measurement. Through survival analysis we examined predictors of virologic failure (2HIV-RNA ≥400 c/l while on cART) including CD4 count and HIV duration at initiation. We also estimated the cumulative probabilities of failure and drug resistance (from the available HIV nucleotide sequences) for early initiators (cART within 12 months of seroconversion).

**Results:**

Of 1075 starting cART at a median (IQR) CD4 count 272 (190,370) cells/mm^3^ and HIV duration 3 (1,6) years, virological failure occurred in 163 (15%). Higher CD4 count at initiation, but not HIV infection duration at cART initiation, was independently associated with lower risk of failure (p=0.033 and 0.592 respectively). Among 230 patients initiating cART early, 97 (42%) discontinued it after a median of 7 months; cumulative probabilities of resistance and failure by 8 years were 7% (95% CI 4,11) and 19% (13,25), respectively.

**Conclusion:**

Although the rate of discontinuation of early cART in our cohort was high, the long-term rate of virological failure was low. Our data do not support early cART initiation being associated with increased risk of failure and drug resistance.

## Introduction

Since combination antiretroviral therapy (cART) was introduced in the mid-1990s there has been a trend towards initiating it earlier in the course of HIV disease. Some major treatment guidelines currently recommend initiation when CD4 cells count drops below 350 cells/mm^3^ while others at CD4 cell count below 500 cells/mm^3^ or in all patients with diagnosed HIV infection (e.g. [1-4]). Moreover, the “test and treat” approach of testing whole populations for HIV followed by immediate initiation of cART for all infected, regardless of CD4 cell count, has gained stimulus as a potential tool for the prevention of onward HIV transmission. Such an approach would result in greater numbers initiating cART in early infection [5-7].

A potential disadvantage of early cART initiation, however, is risk of failure and subsequent development of drug resistance. This is driven by concern that asymptomatic individuals with low absolute risk of clinical disease, who may not be ready to commit to life-long cART, might be at raised risk of developing drug resistance due to sub-optimal adherence. This concern was not supported by a number of studies showing that higher CD4 cell count at cART initiation was associated with lower risk of drug resistance [8-10]. Nonetheless, there are no published data on the long-term risk of developing resistance following early treatment initiation, or on the role of duration of HIV infection at initiation as a predictor of virological failure or of resistance. These data are important to inform the debate on when to initiate treatment.

We have, therefore, used data from the UK Register of HIV Seroconverters to estimate the risk of virological failure while on cART as a proxy of development of drug resistance in patients who initiated cART in early HIV infection, and to assess duration of infection at cART initiation as a predictor of failure. In a subset of patients, for whom HIV nucleotide sequences were available, we also examined the role of HIV infection duration on the long-term risk of drug resistance. Our study hypothesis is that shorter duration of HIV infection at cART initiation is associated with increased risk of virological failure and development of drug resistance.

## Methods

### Patients

The UK Register of HIV Seroconverters is a cohort established in October 1994 with the initial aim of studying the natural history of HIV infection [11]. Eligible patients are aged >15 years with a positive HIV test result within 3 years of a negative HIV antibody test result, or with laboratory evidence of acute infection. Since 2004 the eligibility criteria were narrowed to include only individuals with an HIV test interval of ≤12 months although those already enrolled remained under follow-up. In 2011 the eligibility criteria were widened to allow the follow-up of perinatally-infected children originally enrolled in the Collaborative HIV Paediatric Study (CHIPS) [www.chipscohort.ac.uk] once they reached >15 years of age. Data are collected at enrolment and annually thereafter, through Clinical Report Forms at clinical centres, and include demographic information, laboratory measurements (including CD4 count and HIV-RNA), details of all antiretroviral therapy prescribed, clinical events and vital status. We used the data updated in September 2011 excluding perinatally-infected children.

We included patients enrolled in the UK Register of HIV Seroconverters who initiated cART aged >15 years, while naive at cART initiation and with at least one CD4 count in the 6 months before initiation, and at least one HIV-RNA measurement >6 months after initiation. We also identified the subgroup of patients who had initiated cART early, i.e. within 12 months of HIV seroconversion.

Failure was defined as the first of two consecutive HIV-RNA measurements ≥400 copies/mL after 6 months of cART initiation while the patient was known to be on at least one antiretroviral drug. Episodes of HIV-RNA ≥400 copies/mL occurring while the patient was not on ART were not considered as virological failure as they were likely to be a consequence of stopping cART rather than virological failure. cART was defined as a combination of at least three drugs from at least two classes, or at least three nucleoside reverse-transcriptase inhibitors, one of which was tenofovir or abacavir.

### Statistical analyses

Follow-up started at date of cART initiation and ended at last recorded HIV-RNA measurement.

Univariable and multivariable Cox proportional hazard models were then used to examine the association between failure and duration of infection at cART initiation since the estimated date of HIV seroconversion (0-3, 4-12, >12 months) adjusting for the potential effects of: age, and CD4 count (natural log scale) at cART initiation, sex, risk group, class of initial cART, calendar year of initiation (<2000 vs. ≥2000), and diagnosis during acute HIV infection (HIV test interval <30 days/ laboratory evidence of acute infection vs. longer test interval). The choice for calendar year cut-off reflected the introduction of boosted protease inhibitors (PIs). Models using fractional polynomials indicated a non-linear age effect on survival and two age groups based on median age at seroconversion (<36 and ≥36 years) were, therefore, used [12]. For simplicity, results are presented from a reduced model for risk of failure including CD4 count, and duration of infection at cART initiation as well as the other variables significant at the 10% level in the univariate analysis. To explore if CD4 count was an independent predictor of risk of failure after accounting for virological status, we refitted the final model adjusting for HIV-RNA (log 10 scale) at cART initiation. To better understand patterns of discontinuation, Kaplan-Meier methods were used to estimate the risk of treatment interruption, defined as at least 30 days with no antiretroviral therapy, according to CD4 count and HIV duration at cART initiation.

For the subset of patients for whom we were able to match HIV nucleotide sequences in the UK Drug Resistance Database [13], we estimated the predictors of detection of one or more drug resistance mutations using the same methods as for virological failure. Drug resistance mutations were defined using the Stanford HIV drug resistance algorithm which assigns a drug resistance susceptibility score to each drug. The nucleotide sequences are scanned for the presence of mutations and the drug specific scores are summed across all mutations in the sequence. Based on the total score, drug susceptibility is classified as “sensitive” (score 1), “potential low-level resistance” (score 2), “low-level resistance” (score 3), “intermediate resistance” (score 4) and “high-level resistance” (score 5). In our analysis, detection of drug resistance was defined as a score 4 or 5 to at least one drug [14]. As not all patients in the UK Register were systematically tested for drug resistance before cART initiation, resistance tests done pre-ART were ignored.

Finally, we estimated the long-term probability of drug resistance in patients who initiated cART within 12 months of HIV seroconversion using Kaplan-Meier methods to estimate the cumulative probability of i) detection of one or more drug resistance mutations from the available nucleotide sequences and ii) virological failure.

We performed the following sensitivity analyses to check the robustness of results. The data were re-analysed using fractional polynomials for duration of HIV infection and/or using categories for calendar year of cART initiation <2001 vs. ≥2001 [13]. We further included Stanford low-level resistance (score 3) in the definition of drug resistance. Finally, to account for the change in inclusion criteria from 3 to 1 year HIV test interval, we reran the analyses using categories for calendar year of cART initiation <2004 vs. ≥2004 and restricting to patients who initiated cART ≥2004.

### Ethics statement

The UK Register has research ethics approval 04/Q2707/155. Written informed consent was obtained from all patients enrolled after 2005. The Ethics committee did not require to go back and get written consent from everyone already enrolled at that stage. No incentives were given to patients to participate the study.

## Results

### Baseline characteristics

**Figure 1:**
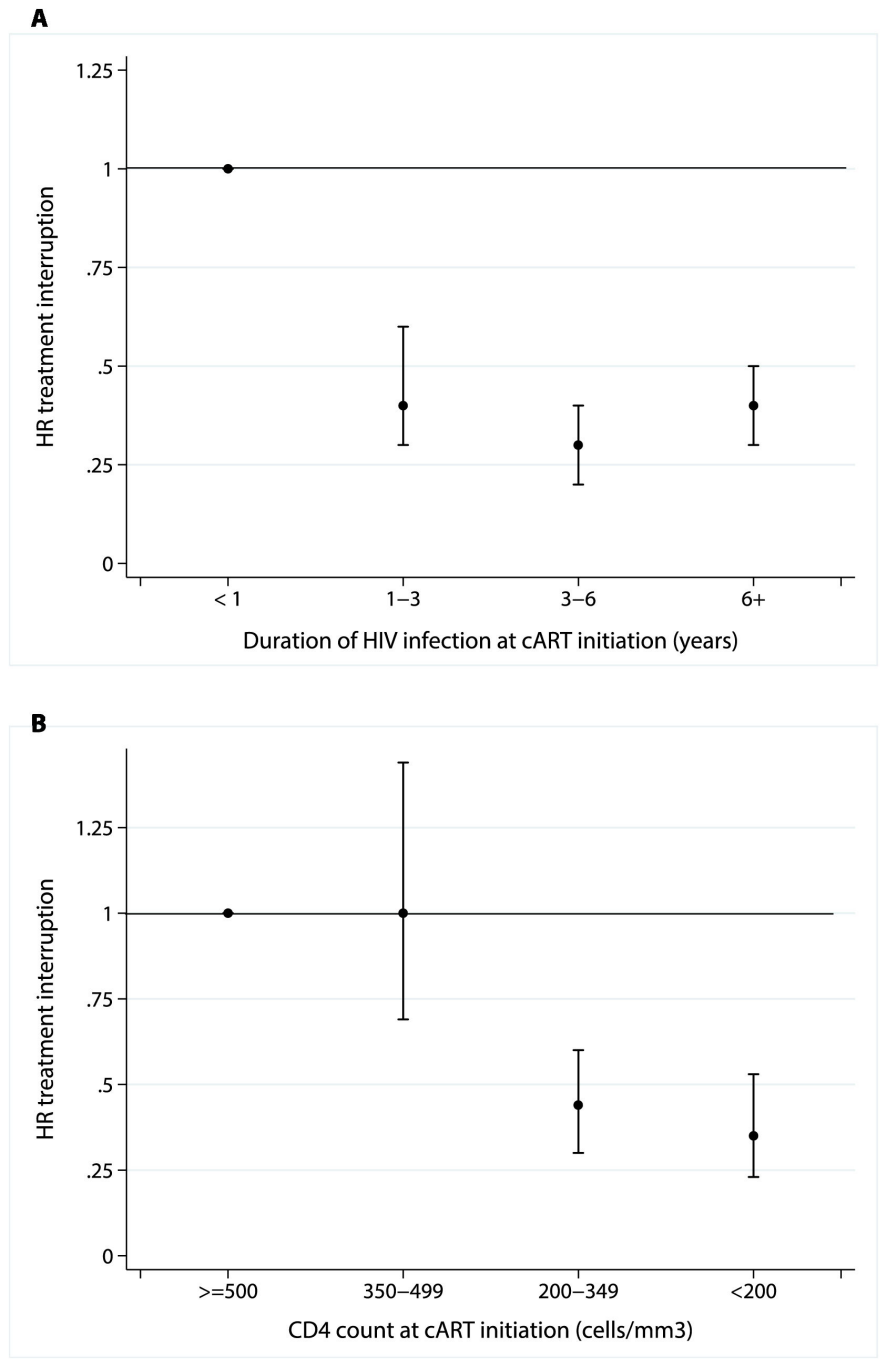
Hazard ratio (HR) k of treatment interruption by duration of HIV infection (as time between HIV seroconversion and cART initiation) and CD4 count at cART initiation.

Of the 2826 patients enrolled in the UK Register of HIV Seroconverters, 1751 were excluded as follows: 1066 did not received cART, 468 were not ART-naive at cART initiation, 134 had no CD4 recorded in the 6 months before cART initiation and 83 had no HIV-RNA after 6 months of cART initiation. The 1075 patients included were mostly homosexual men who started with an NNRTI-based cART regimen (Table 1). Median CD4 count [IQR] at cART initiation was 272 [190,370] cells/mm^3^. Discontinuation of treatment rates were high with 264 (25%) discontinuing cART at least once. Among those who discontinued, 81 restarted treatment after a median [IQR] of 7 [3,15] months. The Kaplan-Meier cumulative probability of discontinuing treatment was 13% (95% CI 11%,15%) and 32% (28%,35%) at 1 and 8 years after cART initiation, respectively. Patients with higher CD4 count and shorter HIV duration at cART initiation were more likely to interrupt treatment (both p log-rank tests <0.001) (Figure 1).

**Table 1:** Baseline characteristics of 1075 seroconverters initiating cART and included in analysis and of 230 early cART initiators, i.e. within 12 months of the estimated date of HIV seroconversion.

	**Overall**		**Early cART initiation**	
	N 1075		N 230	
*Male, N(%*)	1009	94%	213	93%
*Risk group, N %*				
Sex between men	948	88%	199	87%
Sex between women and men	90	8%	25	11%
Injecting drug use	29	3%	2	1%
Unknown/Other	8	10%	4	2%
*Calendar year of cART initiation, median IQR*	2003	[2000,2007]	2003	[2000,2006]
*Age at cART initiation (years*)*, median IQR*	36	[30,42]	33	[27,39]
*HIV test interval (days*)* *, median IQR*	250	[100,470]	113	[8,244]
*HIV test interval <12 months, N %*	713	66%	208	90%
*Laboratory evidence of acute infection, N %*	88	8%	47	20%
*Duration of HIV infection cART initiation (years since date of HIV seroconversion*)*, median IQR*	3.1	[1.2,6.0]	0.4	[0.2,0.6]
*CD4 cell count at cART initiation (cells/mm* ^*3*^)*, median IQR*	272	[190,370]	390	[233,538]
*CD4 cell count at cART initiation ≥500 cells/mm* ^*3*^ *, N %*	112	10%	64	28%
*CD4 cell count at cART initiation ≥350 cells/mm* ^*3*^ *, N %*	317	3%	128	56%
*HIV-RNA at cART initiation (log*_*10*_ * copies/mL*)*, median IQR*	5	[4.5,5.5]	5.2	[4.7,5.7]
*Class of initial cART*				
NNRTI	710	66%	125	54%
unboosted PI	134	12%	30	13%
boosted PI	174	16%	66	29%
3 NRTI	41	4%	6	3%
3 Class	16	1%	3	1%
*Length of follow-up (years between cART initiation and last HIV-RNA*)*, median [IQR*]	5	[2,8]	5	[2,8]
*Discontinued treatment during follow-up* *^* *, N %*	264	25%	97	42%
*Time from cART initiation to first episode of discontinuation (months*)*^* *, median [IQR*]	12	[3,31]	7	[3,13]
*Duration of first episode of discontinuation (months*)*^* *, median [IQR*]	14	[5,32]	29	[13,45]
*Restarted cART after the first episode of discontinuation, N %*	251	95%	94	96%
*Duration of second episode of discontinuation (months*)*^* *, median [IQR*]	8	[3,19]	8	[3,54]
*At least one episode of virological failure, N %*	163	(15%)	33	(14%)
*At least one nucleotide sequence available, N %*	233	(22%)	76	(33%
*Drug resistance to at least 1 drug detected, N %*	82	8%	13	6%
*Crude rate of resistance overall, events/100 person years, 95 % CI*	1.4	(1.1,1.7)	1.1	(0.6,1.8)
*Crude rate of resistance while on cART, events/100 person years, 95% CI*	1.3	(1.1,1.7)	1.1	(0.5,2.1)

* Time elapsing between the last negative and first positive HIV antibody test

^ Treatment discontinuation was defined as at least 30 days without any antiretroviral drugs

Two hundred and thirty patients started cART within 12 months of HIV seroconversion with median CD4 count of 390 [233,538] cells/mm^3^ at initiation. Treatment discontinuation in this subgroup was more common (42%) compared to the overall included patients after median cART duration of 7 [3,13] months, reflecting a common practice during the study period of prescribing short-course therapy in primary HIV infection.

### Effect of HIV infection duration on risk of virological failure

One hundred sixty three (15%) experienced at least one episode of virological failure during a follow-up of 4524 person years (PY). The crude rates of failure were 3.6 (95% CI 3.1,4.2) events per 100 PY overall and 3.7 (2.0,6.3), 3.4 (2.0,5.3) and 3.6 (3.0,4.3) for individuals with HIV infection duration at cART initiation ≤3 months, 4-12 months and >12 months, respectively.

A summary of the univariate and multivariable Cox models of the association of covariates, i.e. characteristics at cART initiation, on the risk of failure is presented in Table 2. While higher CD4 count, age ≥36 years at cART initiation and calendar period ≥2000 were independently associated with lower risk of failure, we found no evidence of an effect of duration of HIV infection. Patients who started cART in or after 2000 had significantly lower hazard of experiencing virological failure compared to those who started before this year (hazard ratio (HR) 0.29, 95% CI 0.21,0.39, p≤0.001). We did not find any other significant effect of the explored covariates on the risk of virological failure. While there was evidence that unboosted PI-based initial cART and presentation during acute infection were associated with a higher risk of failure in the univariate analysis, these associations disappeared after adjustment for CD4 count and calendar year of cART initiation. When we refitted the multivariable model to include HIV-RNA at cART initiation, neither HIV-RNA nor CD4 count at cART initiation were significantly associated with risk of failure (HR 1.08 (95% CI 0.86,1.37), p=0.498 and 0.85 (0.69,1.06), p=0.148, respectively). We found similar results when using calendar year of cART initiation <2001 vs. ≥2001 and <2004 vs. ≥2004 and restricting to patients who initiated cART ≥2004.

**Table 2:** Predictors of risk of virological failure following cART initiation for 1075 patients.

	***UnivariableCoxmodels***					***MultivariableCoxmodel***			
	*Hazard Ratio*	*95% CI*		*P-value* ^*^		*Hazard Ratio*	*95% CI*		*P-value* ^*^
*Duration at cART initiation (months since date of HIV seroconversion*)									
≤3	1			0.933		1			0.592
4-12	0.89	(0.43,	1.82)			0.76	(0.36,	1.58)	
≥12	0.86	(0.53,	1.73)			0.72	(0.38,	1.36)	
*CD4 count at cART initiation* (ln cells/mm^3^ increase)	0.84	(0.68,	1.02)	0.087		0.82	(0.69,	0.98)	0.033
*Age at cART initiation* (years)									
<36	1					1			0.08
≥36	0.64	(0.47,	0.87)	0.005		0.75	(0.55,	1.03)	
*Calendar year of cART initiation*									
<2000	1					1			<0.001
≥2000	0.29	(0.21,	0.39)	<0.001		0.32	(0.22,	0.47)	
*Identification during acute infection*	0.71	(0.52,	0.97)	0.030		0.82	(0.59,	1.14)	0.236
*Female*	0.49	(0.22,	1.20)	0.120					
*Risk group*									
Sex between men	1			0.183					
Injecting Drug users	0.59	(0.19,	1.86)						
Sex between a woman and a man	0.58	(0.29,	1.19)						
*Class of initial cART*									
NNRTI	1			<0.001		1			0.639
Unboosted PI	2.68	(1.87,	3.84)			1.18	(0.77,	1.79)	
Boosted PI	1.02	(0.62,	1.68)			1.43	(0.76,	2.69)	
Other	1.48	(0.79,	2.78)			1.16	(0.70,	1.94)	

* P-values for Wald test for continuous and binary variables and Wald test for heterogeneity for categorical variables

### Effect of HIV infection duration on risk of resistance

There were 270 episodes of virological failure and, of these, 36 (13%) were followed by a genotyping test within 12 months. Two hundred and thirty-three patients (21%) had a least one HIV nucleotide sequence during follow-up, of whom 82 (36%) had evidence of drug resistance, 72 while on cART. Median [IQR] HIV-RNA in the 3 months before the genotypic resistance test was 5.0 [4.5,5.5] log_10_ copies/mL. The crude drug resistance rate was 1.4 (95% CI 1.1,1.7) events per 100 person-years (PY) of follow-up overall (including periods off ART), and 1.3 (95% CI 1.1,1.7) events/100 PY on cART. The most common resistance mutations were non-nucleoside reverse transcriptase inhibitors (NNRTI) (49 patients) and nucleoside reverse-transcriptase inhibitor (NRTI) drugs (64 patients), while resistance to PIs and nucleotide transcriptase inhibitors (NtRTI) were less common (14 and 19 patients, respectively). Similarly to the virological failure analysis, we found no evidence of an association between duration of HIV infection at cART initiation and development of drug resistance when this was treated as the outcome in the unadjusted and adjusted analyses. Moreover, we found similar significant effects for the other covariates with the adjusted hazard ratio for cART initiation ≥2001 vs <2001 being 0.53 (0.30,0.96).

### Long term probability of drug resistance in patients who initiated cART early

**Figure 2:**
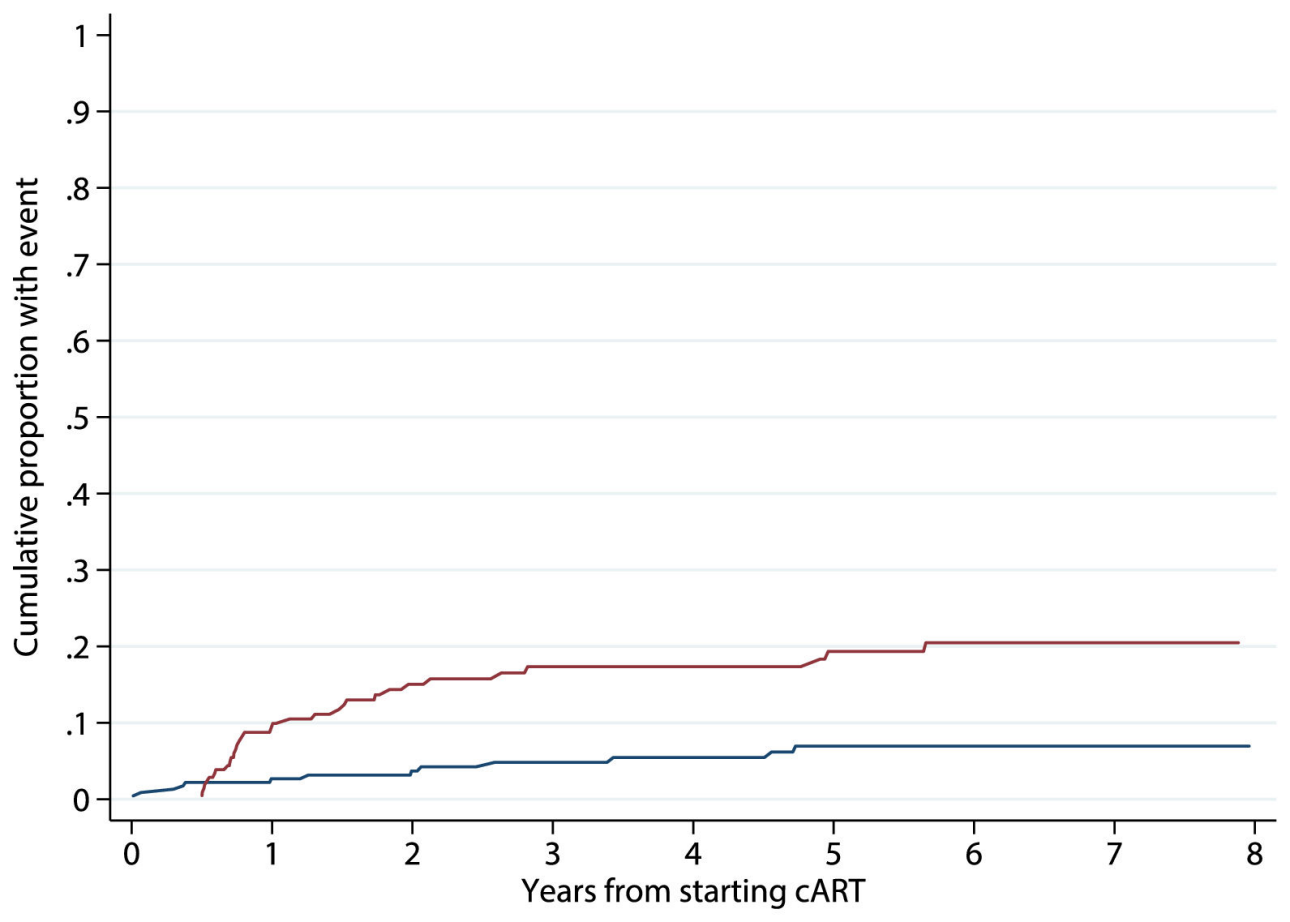
Kaplan-Meier curves for the cumulative probabilities of detection of HIV drug resistance-associated mutations (dashed line) and of failing treatment (HIV-RNA≥400 copies/mL while on treatment) in early cART initiators, i.e. within 12 months of the estimated date of HIV seroconversion.

Of the 230 patients who initiated cART early, 106 (45%) had a resistance test while ART-naïve, of whom 6 (6%) had evidence of transmitted drug resistance. Thirteen developed at least one drug resistance-associated mutation based on the 76 available HIV-RNA nucleotide sequences, 10 while known to be on cART. The estimated crude drug resistance rate was 1.1 (95% CI 0.6,1.8)/100 PY overall (including periods off ART) and 1.1 (95% CI 0.5,2.1)/100 PY while on cART. Thirty-three (14%) of 230 early initiators experienced at least one episode of virological failure during 936 PY follow-up. Figure 2 depicts the cumulative probabilities of detection of drug resistance mutations and of virological failure by time since cART initiation. By 8 years following cART initiation 7% (95% confidence intervals 4%,11%) are estimated to have evidence of drug resistance, and 19% (13%,25%) of previous or current virological failure. Results did not materially change when Stanford score 3 was considered as indicative of drug resistance.

## Discussion

To our knowledge, this is the first study to examine the role of HIV infection duration at cART initiation on the risk of virological failure and development of drug resistance, and to provide estimates of the risk of these two events for patients initiating cART within 12 months of HIV seroconversion. Although, as expected, we found that higher CD4 count at cART initiation was associated with lower risks of failure and resistance, we found no evidence to suggest that this was the case for infection duration at the time cART is initiated. Of note, however, we also found that, for patients initiating cART within 12 months of infection, the proportion developing resistance by 8 years lay between 7% and 19% comparable to estimates reported for individuals initiating cART during chronic infection [9]. Furthermore, the rate of detection of drug resistance in the early initiators was 1.1 (95% CI 0.6,1.8) /100 PY, similar to the rate reported by the Swiss HIV cohort study based on seroprevalent patients starting cART, i.e. individuals with unknown date of HIV infection [15].

The rate of cART discontinuation among those initiating within 12 months of HIV infection and/or with CD4 count >350 cells/mm^3^ was high in our cohort (Figure 1). This is because short-course cART in primary HIV infection was a common treatment strategy in the United Kingdom until recently (median [IQR] cART duration being 7 [3,13] months). Treatment interruption, at least in chronic infection, is no longer recommended [16] and the recent results from SPARTAC have shown only a modest delay in disease progression after a 48-week cART course in primary HIV infection [17]. Nevertheless, this high rate provides a good illustration of what might be expected from frequent treatment discontinuation if asymptomatic HIV-positive individuals start cART early in the course of HIV disease.

Our study does not therefore provide any evidence of early cART initiation being associated with increased risk of virological failure and development of drug resistance after cART initiation. These findings should be interpreted with caution, however, as they do not address the question of when to start cART, because of the dangers of inferring treatment effects from observational data [18,19]. In this non-randomised setting, the effect of HIV infection duration, or CD4 count at cART initiation, on the risk of failure could be partially explained by confounding if the reasons for starting treatment early, rather than delaying until chronic infection, were also associated with the risk of developing resistance and failure. In addition, we studied risk of resistance using cART initiation as baseline. From the point of view of a person in whom the decision whether to initiate cART is being made, any delay in initiation represents a delay in the time before they put themselves at risk of treatment-acquired resistance. The question, therefore, still remains as to whether it is beneficial to initiate cART early and can only be addressed within randomised clinical trials. These should be designed to also estimate the prevalence and incidence of clinically-relevant drug resistance mutations over relatively long follow-up time. The ongoing START trial has such an aim and is currently recruiting [20].

Other studies have examined predictors of resistance [8-10,21-23] and failure [24,25] in seroprevalent cohorts. Our finding that higher CD4 count at cART initiation was an independent predictor of risk of failure and drug resistance was supported by two of these studies [9,10,24]. In contrast with published findings from observational studies and clinical trials, we found no evidence that the use of boosted PI-based cART was associated with a lower risk of failure and resistance compared to NNRTI [9,21-27].

Our study has a number of limitations. First, treatment discontinuation was based on prescribed medication rather than actual drugs taken and we may, therefore, have underestimated the frequency of interruption. Second, a non-negligible proportion of patients in our study started combinations that are less potent than those in current use in the UK. HIV genotyping, as is currently recommended and used in routine practice in the UK, is likely to further reduce the risk of virological failure and resistance. Third, we were able to match a low proportion of treated individuals experiencing failure with HIV nucleotide sequences. Estimates of failure due to the development of drug resistance are, therefore, likely to lie between our estimates of resistance (from nucleotide sequences) and virological failure (from HIV-RNA results). Moreover, given that not all patients were genotyped shortly following infection, some resistance-associated mutations may, in fact, have been transmitted. It is, therefore, possible that our estimates of risk of resistance are somewhat pessimistic. Fourth, although it is recommended that HIV testing be offered at least annually to at risk individuals in the UK [28], is confidential and free of charge, rates of previous HIV testing are low among newly-diagnosed individuals [29]. Individuals identified as seroconverters, and enrolled into the UK Register, are likely, therefore, to not be representative of all those infected. Rates of virological failure reported here may not, therefore, be generalizable, although it is worth noting that CASCADE has reported no evidence of a difference in HIV disease progression and outcome between seroconverters and those with prevalent HIV infection (individuals without estimated dates of HIV seroconversion [30]. In any case, it is not possible, by definition, to assess the role of HIV infection duration without using data from seroconverters. Finally, individuals included in our study are from a resource-rich country and results may not apply to other settings.

In conclusion, we found no evidence to suggest that initiation of cART in early HIV infection is predictive of increased risk of virological failure while on treatment and drug resistance. Moreover, the long-term risk of failure in patients who initiated early, was relatively low. Our findings should provide comfort to patients and their clinicians that early initiation of cART is unlikely to have a detrimental impact on the long-term risk of drug resistance. Nevertheless, data from randomised clinical trials are needed to shed light on the long term clinical benefits and side effects, including development of drug resistance, of early versus delayed cART initiation.
